# Modern paradigms for prostate cancer detection and management

**DOI:** 10.5694/mja2.51722

**Published:** 2022-10-02

**Authors:** Isabella SC Williams, Aoife McVey, Sachin Perera, Jonathan S O’Brien, Louise Kostos, Kenneth Chen, Shankar Siva, Arun A Azad, Declan G Murphy, Veeru Kasivisvanathan, Nathan Lawrentschuk, Mark Frydenberg

**Affiliations:** ^1^ Royal Melbourne Hospital Melbourne VIC; ^2^ Peter MacCallum Cancer Centre Melbourne VIC; ^3^ University of Melbourne Melbourne VIC; ^4^ Singapore General Hospital Singapore Singapore; ^5^ University College London London United Kingdom; ^6^ Monash University Melbourne VIC; ^7^ Cabrini Institute, Cabrini Health Melbourne VIC

**Keywords:** Prostatic neoplasms, Prostate, Chemotherapy, Radiation oncology, Radiotherapy, Surgical oncology

## Abstract

- Early detection and management of prostate cancer has evolved over the past decade, with a focus now on harm minimisation and reducing overdiagnosis and overtreatment, given the proven improvements in survival from randomised controlled trials.
- Multiparametric magnetic resonance imaging (mpMRI) is now an important aspect of the diagnostic pathway in prostate cancer, improving the detection of clinically significant prostate cancer, enabling accurate localisation of appropriate sites to biopsy, and reducing unnecessary biopsies in most patients with normal magnetic resonance imaging scans.
- Biopsies are now performed transperineally, substantially reducing the risk of post‐procedure sepsis.
- Australian‐led research has shown that prostate‐specific membrane antigen (PSMA) positron emission tomography/computed tomography (PET/CT) has superior accuracy in the staging of prostate cancer than conventional imaging (CT and whole‐body bone scan).
- Localised prostate cancer that is low risk (International Society for Urological Pathology [ISUP] grade 1, Gleason score 3 + 3 = 6; and ISUP grade group 2, Gleason score 3 + 4 = 7 with less than 10% pattern 4) can be offered active surveillance, reducing harms from overtreatment.
- Prostatectomy and definitive radiation remain the gold standard for localised intermediate and high risk disease. However, focal therapy is an emerging experimental treatment modality in Australia in carefully selected patients.
- The management of advanced prostate cancer treatment has evolved to now include several novel agents both in the metastatic hormone‐sensitive and castration‐resistant disease settings. Multimodal therapy with androgen deprivation therapy, additional systemic therapy and radiotherapy are often recommended.
- PSMA‐based radioligand therapy has emerged as a treatment option for metastatic castration‐resistant prostate cancer and is currently being evaluated in earlier disease states.

Australia has among the world’s highest incidence of prostate cancer,^1^ with about one in six men diagnosed by the age of 85 years. At present, the prostate cancer 5‐year survival rate in Australia is 96%, which has significantly improved from 60% in the previous 30 years.^2^

Technological advances have ushered a paradigm shift in the understanding, detection and management of prostate cancer in Australia, transitioning from overdetection and overtreatment to the use of sophisticated detection and treatment methods focused on harm minimisation. As such, there are fewer unnecessary biopsies, safer biopsy experiences, and a higher likelihood of active surveillance for low and intermediate risk disease, reducing harms.

Recent research and technological advancements have addressed disease progression in different risk groups.^3^, ^4^ Multimodal early detection, with serum prostate‐specific antigen (PSA) and multiparametric magnetic resonance imaging (mpMRI), has reduced the need for biopsy, with a transperineal approach reducing post‐procedural sepsis rates. More accurate staging with prostate‐specific membrane antigen (PSMA) positron emission tomography/computed tomography (PET/CT) avoids futile local interventions, and treatment options for localised disease now include active surveillance reducing harms such as incontinence, erectile dysfunction, and radiation cystitis and proctitis. Patient characteristics, including symptom profile, comorbid conditions, and lifestyle, are the central focus when deciding individualised management.^5^, ^6^, ^7^ Building upon a review published in 2020,^7^ this article emphasises the ongoing need for screening with PSA as a critical risk‐assessment tool, highlighting that PSA has become a triage tool for mpMRI, and mpMRI a triage test before biopsy. This review explores the latest evidence for PSMA PET/CT for staging of prostate cancer and covers the best evidence‐based management of localised and advanced prostate cancer. It addresses and summarises recent prostate cancer screening and management research updates and focuses on harm minimisation. We searched the online databases PubMed, Google Scholar and the Cochrane Library, with an emphasis on prospective multicentre studies published between 2018 and 2022. We collated local and international guidelines, published data, and expert reviews.

## Early detection of prostate cancer: advances to reduce overdiagnosis

Population screening for prostate cancer has remained controversial since the implementation of Medicare Benefits Schedule (MBS)‐subsidised PSA testing in 1989. Although screening with PSA and digital rectal examination (DRE) remain critical in risk assessment and initial detection,^5^, ^8^ overdetection and overtreatment have been mitigated with improved diagnostic pathways and magnetic resonance imaging (MRI)‐directed targeting.^5^ Australia is a pioneer in using mpMRI and PSMA PET/CT, benefiting significantly from government‐based reimbursements. Federal funding for prostate MRI has improved access to guideline‐based care for men living regionally or in low socio‐economic areas.^9^

The move from ten‐ to 12‐core transrectal ultrasound (TRUS)‐guided prostate biopsy to MRI‐directed targeting plus systematic transperineal prostate biopsy has resulted in safer biopsy procedures, improving diagnostics and accuracy^5^ (Box 1). Following biopsy, staging of prostate cancer has had a shift from conventional CT and bone scan to the use of PSMA PET/CT, which allows differentiation of diagnosis from localised prostate cancer (if cancer confined to the prostate) to advanced prostate cancer (if spread seen to lymph nodes or other organs).

Box 1 **Change in the diagnosis of prostate cancer over time^5^, ^10^, ^11^, ^12^, ^13^**

**Figure:**
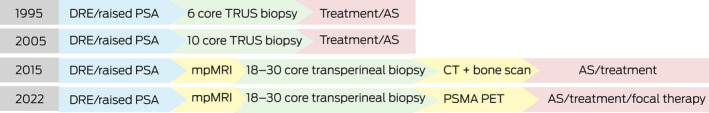
AS = active surveillance; CT = computed tomography; DRE = digital rectal examination; mpMRI = multiparametric magnetic resonance imaging; PET = positron emission tomography; PSA = prostate‐specific antigen; PSMA = prostate‐specific membrane antigen; TRUS = transrectal ultrasound.

### Prostate‐specific antigen screening

PSA screening continues as a critical risk‐assessment tool in triaging select men for further investigation, but it is not recommended nor routinely performed in all asymptomatic men.^5^, ^6^ Informed decision making is imperative, considering age, life expectancy, and risk factors, including family history.^5^ If used inappropriately, PSA screening can lead to excessive interventions, as levels may rise in benign conditions (including benign prostatic hyperplasia, prostatitis, manipulation of the prostate), potentially leading to unnecessary biopsies and overtreatment if all cancers are actively treated.^5^

The European Randomized Study of Screening for Prostate Cancer (ERSPC) data found, after 16 years of follow‐up, that 570 men would need PSA screening to prevent one prostate cancer‐related death, numbers similar to breast cancer screening programs using mammography.^14^ The extended 22‐year follow‐up Göteborg randomised prostate cancer screening trial — one of the arms of the ERSPC study — found a decreased overdiagnosis rate, with a drastically reduced number needed to diagnose (nine men) and a relative risk reduction for prostate cancer mortality of 41%.^15^ It was clear that men needed to have at least a 7–10‐year life expectancy to achieve these survival benefits, as the survival curves until that point directly overlap. The United States‐based Prostate, Lung, Colorectal, and Ovarian (PLCO) cancer screening trial (19 years of follow‐up) is in contrast to ERSPC, which randomised men between the intervention arm (annual PSA screening for 6 years and annual DRE for 4 years) and the control group. The PLCO trial found no reduction in prostate cancer mortality between the groups.^16^ Unfortunately, this trial had widespread screening contamination in the control arm (a group supposedly non‐screened), with 46% having PSA screening before enrolment and up to 47.5% throughout the trial, making it difficult to conclude that there was no survival benefit with prostate cancer screening as it was comparing opportunistic testing to a formal screening program.^16^

Guidelines approved by the Australian National Health and Medical Research Council (NHMRC) recommend that well informed men aged 50–69 years have PSA screening every 2 years, with further investigations if total PSA level is > 3.0 ng/mL.^6^ Earlier screening is recommended for men with increased risk, including significant family history.^8^ These recommendations align with international guidelines^5^, ^17^, ^18^ (Box 2), which remain at odds with the Royal Australian College of General Practitioners’ (RACGP) Red Book, stating that neither DRE nor PSA testing is recommended for asymptomatic men unless requested following discussion about test risks and benefits.^19^ Given general practitioners commonly order PSA tests, and that the RACGP endorsed the NHMRC guidelines, it is imperative that the RACGP update the Red Book to be consistent with contemporary guidelines and studies, as outdated advice causes confusion. Australian men should be appropriately PSA‐screened, identifying and managing and monitoring disease at an early stage, preventing late diagnosis with poorer prognosis.

Box 2 **International guidelines for PSA screening for prostate cancer^5^, ^8^, ^17^, ^18^**

**Table:** 

Guidelines	Recommendations	Men at increased risk	Not recommended
Prostate Cancer Foundation Australia	Well informed men aged 50–69 years	From age 45 years for men with 2.5–3 times increased risk (eg, a brother diagnosed, particularly if diagnosed at age < 60 years), or age 40 years for those with nine to ten times increased risk (eg, father and two brothers diagnosed)	Men aged > 70 years or those with a life expectancy < 15 years
European Association of Urology	Well informed men aged ≥ 50 years	From age 45 years for men with family history of prostate cancer or men of African descent, and from age 40 years for men carrying *BRCA2* mutations	Men ≥ 70 years or those with a life expectancy < 15 years
American Urological Association	Well informed men aged 55–69 years	For men younger than 55 years at higher risk (African American, family history of metastatic prostate cancer, genetic mutations) screening should be individualised	Men < 40 years, men > 70, or those with < 10–15‐year life expectancy
National Comprehensive Cancer Network	Men aged 55–69 years	From age 45–75 years for average risk patients, and from age 40–75 years for African American patients or those with germline mutations	Men aged < 40 years, men > 70 years, or those with < 10–15‐year life expectancy

### Multiparametric magnetic resonance imaging

The introduction of mpMRI revolutionised prostate cancer diagnosis and is now becoming standard as a triage test before biopsy, and PSA screening has largely become a triage tool for mpMRI. This type of scan reduces the need for biopsy if negative for malignant lesions.^5^, ^20^ In Australia, since the inclusion of MRI in the MBS in 2018, men meeting the criteria can access MRI without out‐of‐pocket costs.^21^ Guidelines now include MRI in the diagnostic pathway,^5^ and mpMRI is cost‐effective in the prostate cancer investigatory pathway.^22^ Currently in Australia, mpMRI is performed following a DRE suspicious for prostate cancer if:

- two PSA tests performed within an interval of 1–3 months are > 3.0 ng/mL, with a free/total PSA ratio < 25%; or
- two PSA tests performed within an interval of 1–3 months are > 2.0 ng/mL, with a free/total ratio < 25% (for men aged < 70 years and a family history of first degree relative with suspected *BRCA1* or *BRCA2* mutation); or
- two PSA tests performed within an interval of 1–3 months are > 5.5 ng/mL, with a free/total ratio < 25% (for men aged > 70 years).

mpMRI is also government‐subsidised for men on active surveillance, and must be requested by urologists or radiation or medical oncologists for MBS funding.^21^

The European Association of Urology (EAU) recommends mpMRI before prostate biopsy in biopsy‐naïve men, and if positive (Prostate Imaging Reporting and Data System [PI‐RADS] ≥ 3),^23^ it should be followed by targeted biopsy (of index lesion) and systematic sampling biopsy.^5^, ^18^ The EAU supports consideration of omitting biopsy following negative MRI results (PI‐RADS ≤ 2) with low clinical suspicion, and shared patient decision making.^5^, ^24^ Where clinical suspicion persists, mpMRI is recommended before repeat biopsy, with systematic biopsy being performed after a negative mpMRI and targeted biopsy alone after positive mpMRI.^5^

The PROMIS trial supported mpMRI in the diagnostic pathway, providing evidence for diagnostic accuracy of mpMRI in biopsy‐naïve patients.^25^ mpMRI‐targeted biopsy showed greater sensitivity (87%) compared with TRUS‐guided biopsy (60%), and higher negative predictive value (72% *v* 65%) for detecting Gleason score 3 + 4 prostate cancer and above. Twenty‐seven per cent of patients with negative mpMRI could potentially have avoided biopsy.^25^ Negative mpMRI alone is currently insufficient to omit prostate biopsy,^26^ unless patients are prepared to accept a 5–10% false negative rate. Likewise, positive mpMRI alone cannot currently replace biopsy.^25^

The PRECISION trial found mpMRI with or without targeted biopsy, compared with standard biopsy without mpMRI, resulted in fewer unnecessary biopsies and in identification of more clinically significant prostate cancer and fewer clinically insignificant cancers, with fewer biopsy cores taken.^27^ Twenty‐eight per cent of men (in the investigatory arm) with absence of suspicious lesion on mpMRI avoided biopsy. mpMRI‐targeted biopsy diagnosed clinically significant prostate cancer in 38% of patients compared with 28% from TRUS‐guided biopsy.^27^

A standardised reporting system, the PI‐RADS, was introduced in 2012 and updated in 2014.^23^, ^28^ This has improved reporting of prostate mpMRI in determining clinical significance^23^ (Box 3).

Box 3 **Prostate Imaging Reporting and Data System (PI‐RADS)^23^, ^29^**

**Table:** 

Score	Clinical significance	PPV of clinically significant prostate cancer
PI‐RADS 1	Clinically significant cancer is highly unlikely to be present	‐
PI‐RADS 2	Clinically significant cancer is unlikely to be present	‐
PI‐RADS 3	Clinically significant cancer is equivocal	13%
PI‐RADS 4	Clinically significant cancer is likely to be present	40%
PI‐RADS 5	Clinically significant cancer is highly likely to be present	69%

PPV = positive predictive value.

PSMA PET/CT may also play a role in prostate cancer local detection and grading. Maximum standardised uptake value (SUVmax) is a measurement of tracer uptake in tissue for PET imaging. Greater SUVmax on PSMA PET/CT was associated with clinically significant prostate cancer (International Society for Urological Pathology [ISUP] grade group 3–5) on biopsy.^30^, ^31^ The combination of PI‐RADS score and PSMA PET/CT SUVmax provided higher sensitivity and negative predictive value than individually with the addition of PSMA PET/CT alongside mpMRI.^30^ The PRIMARY trial, a recent Australian‐led multicentre trial, evaluated the use of pelvic PSMA PET/CT in the diagnosis of intraprostatic malignancy in men with mpMRI PI‐RADS 2–5. This trial found that mpMRI and PSMA PET/CT combined imaging improved negative predictive value (91%) and sensitivity (97%) for clinically significant prostate cancer.^13^ Future studies will determine whether biopsy may be safely omitted in men with high clinical suspicion of clinically significant prostate cancer with negative combined imaging.^13^ Although PI‐RADS scoring has long been used for MRI, recent research has led to the development of a standardised reporting system for PSMA PET/CT, with research ongoing.^32^, ^33^ PSMA PET/CT may suit men unable to have mpMRI prostate due to metallic foreign bodies or implants, as localisation with PSMA PET/CT can assist lesion targeting at biopsy.

### Transperineal prostate biopsy

The use of transperineal prostate biopsy in preference to TRUS‐guided biopsy has shown a reduced risk of sepsis and hospitalisations, with hospitalisation rates for transperineal prostate biopsy being 0–0.7% compared with 0.5–6.9% for TRUS‐guided biopsy.^34^ Both approaches have acceptable accuracy for mpMRI‐targeted biopsy.^35^

TRUS‐guided biopsy is traditionally performed in consulting rooms or outpatient settings using local anaesthesia, and transperineal prostate biopsy is generally carried out under general anaesthesia, requiring hospital theatre time.^36^ There is progression towards local anaesthesia for transperineal prostate biopsy,^36^ with current evidence supporting preference of the transperineal over the transrectal approach.^5^ In Australia, the MBS has been revised to encourage the use of transperineal prostate biopsy over TRUS‐guided biopsy based on current evidence.^37^

The Gleason score remains the recommended grading system for prostate cancer biopsies, reporting the most extensive pattern plus the highest pattern.^5^ In 2014, to reduce confusion over the clinical difference between Gleason scores 3 + 4 = 7 and 4 + 3 = 7, and to align prostate cancer grading with other carcinoma grading, the ISUP introduced grade groups^5^, ^38^ (Box 4).

Box 4 **International Society for Urological Pathology (ISUP) scoring risk significance^38^**

**Table:** 

ISUP grade group	Gleason score	Risk for clinically significant prostate cancer
1	≤ 6	Low risk
2	3 + 4 = 7	Intermediate favourable risk
3	4 + 3 = 7	Intermediate unfavourable risk
4	8 (4 + 4 or 3 + 5 or 5 + 3)	High risk
5	9–10 (4 + 5 or 5 + 4 or 5 + 5)	Highest risk

The ISUP recently recommended including the presence of cribriform pattern and/or intraductal carcinoma in standard reporting,^39^ which is clinically important due to the correlation with advanced stage, metastasis, and subsequent overall poor prognosis. Studies found men with Gleason score 3 + 4 = 7 without cribriform pattern have similar prognosis to men with Gleason score 3 + 3 = 6, and men with cribriform pattern were more likely to have biochemical recurrence or higher radiotherapy failure after radical prostatectomy.^40^, ^41^

## Staging of prostate cancer using PSMA PET/CT

Formerly, conventional CT and bone scan were the gold standard for prostate cancer staging. Men with localised disease would undergo definitive therapy, either radical prostatectomy or radiotherapy. However, despite optimal treatment, up to 50% of men with high risk localised prostate cancer experienced biochemical recurrence within 5 years.^42^ Many then received treatment guided by imaging modalities with insufficient accuracy to detect non‐localised disease, thus not beneficial.^43^

Recently, Australian investigators have delineated the role of PSMA PET/CT for early prostate cancer staging. One hallmark study, the proPSMA trial, assessed the utility of PSMA PET/CT against standard of conventional CT and bone scan for primary staging.^44^ This prospective randomised phase 3 study across ten Australian centres randomly allocated 302 patients with high risk prostate cancer to receive PSMA PET/CT or conventional CT and bone scan before radical prostatectomy. Investigators reported PSMA PET/CT had 27% greater accuracy than conventional imaging (92% *v* 65%; *P* < 0.0001). PSMA PET/CT had lower radiation exposure (8.2 mSv *v* 19.2 mSv; *P* < 0.001), lower rate of equivocal findings (7% *v* 23%), and overall higher reporter agreement (κ = 0.87 for nodal, and κ = 0.88 for distant metastases). Significantly, upfront PSMA PET/CT staging changed management for 27% of men in the PSMA PET/CT group compared with 5% in the conventional imaging arm.^44^

Overall, proPSMA provides compelling evidence that PSMA PET/CT offers superior accuracy, better informing clinical practice than conventional imaging for newly diagnosed prostate cancer. A health economics review of proPSMA found that PSMA PET/CT was superior in cost ($1140 *v* $1181 respectively) and adjusted for cost per quality‐adjusted life‐year. Furthermore, each additional metastasis detected by PSMA PET/CT resulted in estimated savings of $428 by avoiding unnecessary treatment.^45^ Long term follow‐up studies must assess whether enhanced detection of de novo metastatic disease missed on conventional imaging will improve survival.

The proPSMA trial was instrumental in the EAU 2022 guidelines acknowledging PSMA PET/CT was a more accurate modality for prostate cancer staging.^5^ Following this, the Australian federal government announced that the MBS would fund PSMA PET/CT from 1 July 2022. To our knowledge, Australia is the first public health care system to adopt PSMA PET/CT imaging. Patients with intermediate to high risk prostate cancer on biopsy are eligible for government‐funded PSMA PET/CT (primary staging) and restaging of patients with recurrent prostate cancer (PSA persistence/biochemical recurrence). It is hoped this national funding initiative will remove access barriers and transition PSMA PET/CT from metropolitan research institutions to the broader community.

## Management of localised prostate cancer

The consensus for stratification of prostate cancer follows ISUP grading,^38^ where ISUP grade group 1–2 is low risk (if Gleason score 4, disease < 10%) and ISUP grade group 3–5 is intermediate to high risk (Box 4). Localised disease description encompasses ISUP, PSA and D’Amico risk stratification with tumour, nodes and metastases staging (Box 5).^46^, ^47^

Box 5 **Risk groups for biochemical recurrence in localised and locally advanced prostate cancer^46^**

**Table:** 

Localised			Locally advanced
Low risk	Intermediate risk	High risk	High risk
PSA < 10 ng/mL and GS < 7 (ISUP grade group 1) and cT1‐2a	PSA 10–20 ng/mL or GS 7 (ISUP grade group 2/3) or cT2b	PSA > 20 ng/mL or GS > 7 (ISUP grade group 4/5) or cT2c	Any PSA, any GS (any ISUP grade group) cT3‐4 or cN+

cN + = lymph node positive; cT1‐2a = cancer present but not detectable on digital rectal examination (DRE) or imaging, or is palpable on DRE but is organ‐confined to half or less than half of one lobe of the prostate; cT2b = tumour‐confined to more than one half of one gland of prostate but not both; cT2c = tumour is in both lobes of the prostate but within the prostate capsule; cT3‐4 = locally extensive cancer that penetrates the prostate capsule, or invades into the seminal vesicle, or invades into the bladder neck/rectum/external urinary sphincter; GS = Gleason score; ISUP = International Society for Urological Pathology; PSA = prostate‐specific antigen.

Recently, there has been a shift away from treatment of low risk, localised disease. The descriptor “clinically significant” is widely used for prostate cancer potentially causing morbidity or death, a distinction essential in preventing overtreatment, as most low risk prostate cancer does not require treatment, thus avoiding harmful side effects.^5^ Typically, this less invasive approach applies to ISUP grade group 1 and some ISUP grade group 2 (patients with < 10% Gleason score 4 pattern, favourable PSA and mpMRI findings).^48^ These findings indicate suitability for active surveillance until life expectancy below 10 years.

The ProtecT trial randomly allocated 1643 men with localised prostate cancer to active surveillance, surgery or radiotherapy, and found that, at a median 10‐year follow‐up, death of prostate cancer was low (about 1%), irrespective of the treatment assigned, and all‐cause mortality was low (~10%).^49^ Similarly, the PIVOT trial found prostatectomy did not result in lower all‐cause or prostate cancer‐specific mortality over active surveillance.^50^ Active surveillance has clear benefits for health‐related quality of life. Patient‐reported outcomes in the ProtecT cohort found that prostatectomy had most significant adverse effects on sexual function and urinary continence. Radiotherapy negatively affected bowel function. The effect of radiotherapy on sexual function and urinary frequency recovered by 2–3 years to levels consistent with active surveillance. Sexual and urinary function declined gradually over time in the active surveillance group. There were no significant differences in anxiety, depression or general health between cohorts.^51^

An examination of the Prostate Cancer Outcomes Registry Australia Victoria (PCOR‐Vic) from 2009 to 2016 (3201 patients with low risk prostate cancer) found an increase in conservative management (no active treatment within 12 months of diagnosis) from 52% in 2009 to 73% in 2016, with active surveillance increasing from 33% in 2009 to 67% in 2016.^52^ Other studies using PCOR‐Vic found almost three‐quarters of men on active surveillance did not have follow‐up investigations consistent with standard protocols, which recommend three PSA measurements and one biopsy within 24 months of diagnosis.^53^ Appropriate follow‐up is imperative to ensure men do not miss the opportunity for curative‐intent treatment.^53^

By committing patients to active surveillance, reliance on surgical investigation is reduced and reliance on PSA velocity and MRI increases.^54^ Increasing active surveillance over the past decade has shown lower proportion of men with low risk localised disease progressing to intervention, with a higher quality of life. Studies also found that selected patients with intermediate risk disease benefit from active surveillance.^55^

The advantages of active treatment of localised prostate cancer are most strongly evidenced in intermediate and high risk disease. Prostatectomy remains the gold standard in surgical management to optimise staging and function, often performed with pelvic lymph node dissection and nerve‐sparing surgery. Surgical management includes open radical prostatectomy, laparoscopic radical prostatectomy and, over the past two decades, the introduction of robot‐assisted radical prostatectomy as a minimally invasive option. A Cochrane systematic review found insufficient evidence comparing oncological outcomes, with urinary and sexual quality of life being similar between groups, noting laparoscopic radical prostatectomy or robot‐assisted radical prostatectomy may reduce blood transfusion frequency.^56^

The use of radiotherapy for treatment of localised prostate cancer is increasing. Advances have been rapid and have yielded encouraging results. Radiotherapy is increasingly precise, using image‐guided techniques such as fiducial markers, volumetric modulated arc therapy and cone beam CT. These technologies allowed reduced radiation to surrounding structures, including the bladder and rectum,^57^ with reduction in acute rectal side effects and/or toxicities with hydrogel spacers between prostate and rectum.^58^ Hypofractionated dose escalation in localised disease has provided better disease control with dose‐escalated therapy, shortening treatment duration.^59^, ^60^

As part of counselling for addressing treatment options, it is essential to discuss adjuvant androgen deprivation therapy (ADT), which can be given with radiotherapy for patients with unfavourable intermediate and high risk prostate cancer. Recent long term observational studies showed equivalence in overall survival for prostatectomy versus external beam radiation therapy.^61^

Focal therapy is an emerging, non‐funded treatment modality in Australia, although there is a paucity of high quality evidence to support routine practice. It remains an experimental modality, with EAU guidelines currently recommending it only for intermediate risk disease within a clinical trial setting or a well designed prospective study.^5^, ^62^ Overseas, medium term follow‐up studies showed the 24‐, 60‐ and 90‐month survival at 99%, 97% and 97%, respectively, using high intensity focused ultrasound,^63^ although 70% of patients underwent second round therapy to achieve disease‐free state.^63^ Further modelling is required before non‐inferiority can be demonstrated against prostatectomy and radiotherapy. Other energy sources used in focal therapies include irreversible electroporation, interstitial laser, and cryotherapy. Irreversible electroporation ablates prostate tissue via a high voltage electric current between transperineally inserted electrodes. This method showed promising early quality of life and oncologic outcomes in an Australian study, which found that 78% of patients were disease‐free after initial irreversible electroporation and 90% had failure‐free survival at 3 years.^64^ Management options for localised prostate cancer are summarised in Box 6.

Box 6 Localised prostate cancer (disease contained within prostate)*

**Table:** 

ISUP grade group	Management options
1	Active surveillance if life expectancy > 10 years Watchful waiting if life expectancy < 10 years In higher risk patients (eg, those with *BRCA2* mutations) may still consider radical prostatectomy or radiotherapy
2	Prostatectomy or radiotherapy^†^ Could consider active surveillance in patients with < 10% Gleason pattern 4 with favourable PSA, until life expectancy < 10 years
3	Prostatectomy or radiotherapy^†^
4	Prostatectomy or radiotherapy^†^
5	Prostatectomy or radiotherapy^†^

ISUP = International Society for Urological Pathology; PSA = prostate‐specific antigen.

* Focal therapy remains an emerging experimental modality and is currently only recommended for use in clinical trial setting.

† Discussing androgen deprivation therapy (ADT), which can be given with radiotherapy.

## Advanced prostate cancer

Before 2004, advanced prostate cancer management was limited to ADT alone. The treatment landscape has significantly evolved, with the armamentarium of treatments increasing. Combinational approaches are emphasised and novel therapies are used earlier in the treatment, with practice‐changing trials summarised in Box 7.

Box 7 **Landmark clinical trials in prostate cancer^5^**

**Table:** 

Trial	Intervention	Primary endpoint	Outcomes	Implications for practice
**Metastatic hormone‐sensitive prostate cancer**				
Docetaxel				
STAMPEDE (Arm C) (2016)^65^	ADT ± docetaxel	OS	Median OS 43.1 (ADT) *v* 59.1 (ADT + docetaxel) months (including M1 cohort only) (HR, 0.81) Median OS: low volume* (HR, 0.76) *v* high volume^†^ (HR, 0.81)	Docetaxel is subsidised on the PBS; it should be considered in all fit patients with mHSPC, particularly with high volume disease
CHAARTED (2015)^66^	ADT ± docetaxel	OS	Median OS 47.2 (ADT) *v* 57.6 (ADT + docetaxel) months (HR, 0.72) Median OS: low volume (HR, 1.04) *v* high volume (HR, 0.63)	
Enzalutamide				
ENZAMET (2019)^67^	ADT ± enzalutamide *v* ADT + non‐steroidal anti‐androgen therapy (45% concurrent docetaxel)	OS	Rates of OS at 3 years: 72% (ADT + non‐steroidal AAT) *v* 80% (ADT+ enzalutamide) (HR, 0.67)	Not currently subsidised on the PBS for this indication; patients can choose to self‐fund enzalutamide
ARCHES (2019)^68^	ADT ± enzalutamide (18% had prior docetaxel)	rPFS	Median OS not reached in either group (HR, 0.66) rPFS (HR, 0.39)	
Abiraterone				
LATITUDE (2017)^69^	ADT ± abiraterone	OS, rPFS	Median OS 36.5 (ADT) *v* 53.3 (ADT + abiraterone) months (HR, 0.66) Median rPFS 14.8 (ADT) *v* 33.0 months (ADT + abiraterone) (HR, 0.47)	Not currently subsidised on the PBS for this indication; patients can choose to self‐fund abiraterone
STAMPEDE (Arm G) (2017)^70^	ADT ± abiraterone	OS	Median OS 3.8 (ADT) *v* 6.6 year (ADT + abiraterone) (HR,0.60) Median OS: low volume (HR, 0.54) *v* high volume (HR, 0.59)	
PEACE‐1 (2021)^71^	ADT ± docetaxel *v* ADT + abiraterone ± docetaxel (± local RT)	PFS, OS	Median OS (ADT + docetaxel alone) *v* NR (ADT + docetaxel + abiraterone) (HR; 0.82) rPFS (HR, 0.50)	
Apalutamide				
TITAN (2019)^72^	ADT ± apalutamide (11% had prior docetaxel)	PFS, OS	Median OS 52.2 months (ADT) *v* NR (ADT + apalutamide) (HR, 0.65) rPFS at 24 months: 46.5% (ADT) *v* 68.2% (ADT + apalutamide) (HR, 0.48)	Not currently subsidised on the PBS for this indication
Darolutamide				
ARASENS (2022)^73^	ADT + docetaxel ± darolutamide	OS	Median OS 48.9 (ADT + docetaxel) months *v* NR (ADT + docetaxel + darolutamide) (HR, 0.68)	Not currently subsidised on the PBS for this indication
**Radiotherapy to the prostate**				
STAMPEDE (Arm H) (2018)^74^	ADT ± radiotherapy to the prostate	OS	Radiotherapy improved failure‐free survival (HR, 0.76) but not OS (HR, 0.92) Survival at 3 years: Low volume: 73% (no RT) *v* 81% (RT) High volume: 54% (no RT) *v* 53% (RT) Failure‐free survival at 3 years: Low volume: 33% (no RT) *v* 50% (RT) High volume: 17 (no RT) *v* 18% (RT)	Radiotherapy to the prostate recommended in patients with recently diagnosed metastatic low volume prostate cancer
**Non‐metastatic castration‐resistant prostate cancer**				
Enzalutamide				
PROSPER (2020)^75^	ADT ± enzalutamide	OS	Median OS 56.3 (ADT) *v* 67.0 (ADT + enzalutamide) months (HR, 0.73)	Not currently subsidised on the PBS for this indication
Apalutamide				
SPARTAN (2018)^76^	ADT ± apalutamide	Metastasis‐free survival	Metastasis‐free survival was 16.2 (ADT) *v* 40.5 (ADT + apalutamide) months (HR, 0.28) Median O 59.9 (ADT) *v* 73.9 (ADT + apalutamide) months (HR, 0.78)	Apalutamide is available on the PBS for this indication
Darolutamide				
ARAMIS (2019)^77^	ADT ± darolutamide	Metastasis‐free survival	Metastasis‐free survival was 18.4 (ADT) *v* 40.4 (ADT + darolutamide) months (HR, 0.41) OS at 3 years: 77% (ADT) *v* 83% (ADT + darolutamide) (HR, 0.69)	Darolutamide is available on the PBS for this indication
**Metastatic castration‐resistant prostate cancer**				
Cabazitaxel				
TROPIC (2010)^78^	Cabazitaxel 25mg/m^2^ *v* 12mg/m^2^ mitoxantrone	OS	Median OS: 12.7 (mitoxantrone) *v* 15.1 (cabazitaxel) months (HR, 0.70)	Cabazitaxel is used after a patient has progressed on docetaxel and is PBS‐subsidised for this indication
Abiraterone				
COU‐AA‐301 (2011)^79^	ADT ± abiraterone (post docetaxel)	OS	Median OS 10.9 (ADT) *v* 14.8 (ADT + abiraterone) months (HR, 0.65)	Abiraterone is available on the PBS for this indication; it can be used before or after chemotherapy
COU‐AA‐302 (2013)^80^	ADT ± abiraterone (pre docetaxel)	OS, rPFS	Median rPFS 8.3 (ADT) *v* 16.5 (ADT + abiraterone) months (HR, 0.53) Median OS 30.3 (ADT) *v* 34.7 (ADT + abiraterone) months (HR, 0.81)	
Enzalutamide				
PREVAIL (2014)^81^	ADT ± enzalutamide (pre docetaxel)	OS, rPFS	Median rPFS 5.4 (ADT) *v* 20.0 months (ADT + enzalutamide) (HR, 0.32) Median OS 31 (ADT) *v* 36 (ADT + enzalutamide) months (HR, 0.83)	Enzalutamide is available on the PBS for this indication; it can be used before or after chemotherapy
AFFIRM (2012)^82^	ADT ± enzalutamide (post docetaxel)	OS	OS 13.8 (ADT) *v* 18.4 (ADT + enzalutamide) months (HR, 0.63)	
Olaparib				
PROFound (2020)^83^	Olaparib *v* abiraterone/enzalutamide (in HRD+ patients only)	rPFS	Cohort A (patients with a *BRCA1*, *BRCA2*, or *ATM* mutation): median rPFS 3.6 (control) *v* 7.4 months (olaparib) (HR, 0.34) Overall cohort: Median rPFS 3.5 (control) *v* 5.8 (olaparib) months (HR, 0.49) Median OS (cohort A): 14.7 (control) *v* 19.1 (olaparib) months (HR, 0.69)	Approved on the PBS for use in mCRPC in patients with a *BRCA1* or *BRCA2* mutation and who have progressed on a prior anti‐androgen
LuPSMA				
TheraP (2021)^84^	^177^Lu‐PSMA *v* cabazitaxel	PSA response rate	PSA response 37% (cabazitaxel) *v* 66% (^177^Lu‐PSMA) (*P* = 0.0001)	^177^Lu‐PSMA‐617 FDA‐approved for use after a taxane and anti‐androgen; not yet TGA‐approved; available in some centres on compassionate access and through clinical trials
VISION (2021)^85^	SOC ± ^177^Lu‐PSMA	OS, rPFS	Median OS 11.3 (SOC) *v* 15.3 (SOC + ^177^Lu‐PSMA) months (HR, 0.62) Median rPFS 3.4 (SOC) *v* 8.7 months (SOC + ^177^Lu‐PSMA) (HR, 0.40)	

AAT = α‐1 antitrypsin; ADT = androgen deprivation therapy; BSC = best standard of care; FDA = Food and Drug Administration; HR = hazard ratio; HRD + = homologous recombination deficiency; mCRPC = metastatic castration‐resistant prostate cancer; mHSPC = metastatic hormone‐sensitive; NR = not reached; OS = overall survival; PBS = Pharmaceutical Benefits Scheme; PFS = progression‐free survival; PSA = prostate‐specific antigen; rPFS = radiographic progression‐free survival; RT = radiotherapy; SOC = standard of care; TGA = Therapeutic Goods Administration.

* Low volume (CHAARTED): less than four bone metastases.

† High volume (CHAARTED): more than four bone metastases (at least one outside the spine or pelvis) ± visceral metastasis.

Metastatic hormone‐sensitive prostate cancer (mHSPC), non‐metastatic and metastatic castration‐resistant prostate cancer (mCRPC) management is reflected in the 2022 EAU guidelines.^5^ These guidelines recommend offering patients diagnosed with mHSPC immediate systemic treatment with ADT, allowing symptom palliation and reducing the risk of harmful disease sequelae, including spinal cord compression and obstructive uropathy. Following the STAMPEDE and CHAARTED trial results, docetaxel chemotherapy was approved for combination use with ADT in mHSPC,^74^ although the benefit from docetaxel appears to largely favour patients with high volume disease.^86^ More recently, second generation anti‐androgens, including abiraterone acetate, apalutamide and enzalutamide, have conferred an additional survival benefit when combined with ADT, and also concurrent with docetaxel as trimodal therapy in select patient groups.^67^, ^87^ Despite this, many Australian men only receive ADT as primary management for mHSPC.^88^ For men with de novo low volume disease, high dose palliative radiotherapy to the prostate also confers an overall survival benefit.^74^

The treatment landscape has vastly changed for mCRPC, defined as biochemical or radiological progression despite castrate serum testosterone levels (< 50 ng/dL or < 1.75 nmol/L). Treatment and sequencing depend on disease volume, patient performance status, comorbid conditions, and prior therapies. For non‐metastatic disease, whereby PSA rises with no visible metastatic disease on conventional imaging, both darolutamide and apalutamide are approved and subsidised, combined with ADT.^5^ Importantly, these trials were not conducted with PSMA PET/CT, and PSMA PET/CT has been shown detect more metastatic spread, thus upstaging diagnosis from localised disease to advanced prostate cancer in many cases. For mCRPC, pivotal trials have shown an overall survival benefit with docetaxel, cabazitaxel (post‐docetaxel) and second generation anti‐androgens, including enzalutamide and abiraterone (pre‐ and post‐docetaxel).^5^ For patients with *BRCA1* or *BRCA2* alterations (somatic or germline), olaparib is approved and available following progression on second generation anti‐androgen.^5^ Radium‐223 is available for patients with bone metastases,^5^ although uptake is low in Australia as there is no current government subsidy.

Australia is a pioneer in PSMA theranostics. Lutetium‐177 (^177^Lu) — a radiolabelled small molecule that binds to PSMA — delivers high radiation doses to prostate cancer cells.^89^ The phase 2 LuPSMA trial in 2018 showed the efficacy (57% of patients had a 50% PSA reduction) of ^177^Lu‐PSMA‐617 in patients with mCRPC who had progressed on all available therapies.^89^ Following this came the practice‐changing TheraP^84^ and VISION^85^ trials, which led to the approval by the United Stated Food and Drug Administration of ^177^Lu‐PSMA‐617 for use in mCRPC after progression following a taxane and a novel anti‐androgen.^5^

In Australia, limited subsidisation by the Pharmaceutical Benefits Scheme (Box 7) means that patient access to some therapies beyond ADT and chemotherapy remains problematic, influencing practice and hindering capacity to maximise survival and clinical outcomes for men with advanced prostate cancer.

## Multidisciplinary care

A multidisciplinary approach (urologists, medical and radiation oncologists, radiologists, nuclear medicine physicians, pathologists, and nurses) in decision making is essential to achieve optimal patient care, especially for patients with advanced prostate cancer.^90^ Multidisciplinary meetings provide effective platforms for discussions and punctual interdisciplinary referrals. Shared management with patients is essential to facilitate informed decisions regarding treatments, management of side effects (both physical and psychosocial), comorbid conditions, symptom progression and palliative care.^91^

Survivorship management guidelines are being developed and improved, with research supporting holistic health care.^91^, ^92^ For optimal health and quality of life after treatment, this multidisciplinary approach remains essential. Survivors need care comprising health promotion, surveillance, new cancer screening, disease progression, symptom management, side‐effect monitoring and treatment, ongoing psychological care, and coordination with their general practitioner.^92^ This requires additional teams including specialised nurses, psychologists, sexual therapists, physiotherapists, and exercise physiologists.

## Conclusion

Over the past decade, we have seen detection and management of prostate cancer evolve, with clear evidence of survival benefit with screening in select patients who have a life expectancy of more than 7–8 years. The focus has been on harm minimisation, reducing overdiagnosis and avoiding overtreatment.

PSA testing remains the initial risk‐assessment tool for general practitioners. Australian and international guidelines on who and when to test have been updated, with plans for further updates in the near future.

Improved diagnostic capabilities from mpMRI and, more recently, PSMA PET/CT (now subsidised by the MBS), has contributed to reducing unnecessary biopsies and providing greater accuracy in biopsy targeting potential clinically significant prostate cancer, thereby reducing harm. Due to advances in these imaging modalities and safer biopsy techniques, risk assessment with appropriate PSA testing should not be delayed. The management of low risk localised prostate cancer has shifted to active surveillance, preventing overtreatment of clinically insignificant prostate cancer and avoiding potential side effects. Management of advanced prostate cancer continues to rapidly evolve. It is imperative for the public and medical community to be aware that the benefit–harm balance in prostate cancer screening programs is now leaning heavily in favour of benefits over harms, and there is a requirement that all groups provide consistent advice and avoid conflicting messages and confusion.

## Open access

Open access publishing facilitated by Monash University, as part of the Wiley ‐ Monash University agreement via the Council of Australian University Librarians.

## Competing interests

Declan Murphy has received reimbursement for participation in advisory boards and delivering lectures from Astellas Pharmaceuticals, Janssen Pharma, Bayer, Ipsen, Ferring, and AstraZeneca. Veeru Kasivisvanathan receives funding from Prostate Cancer UK and the John Black Charitable Foundation for unrelated work. Arun Azad has been a consultant for Astellas, Janssen, Novartis, and Aculeus Therapeutics; has participated in speaker bureaus for Astellas, Janssen, Novartis, Amgen, Ipsen, Bristol‐Myers Squibb, Merck Serono, and Bayer; has received honoraria from Astellas, Novartis, Sanofi, AstraZeneca, Tolmar, Telix, Merck, Serono, Janssen, Bristol‐Myers Squibb, Ipsen, Bayer, Pfizer, Amgen, Noxopharm, Merck Sharpe and Dome, and Aculeus Therapeutics; has served on scientific advisory boards for Astellas, Novartis, Sanofi, AstraZeneca, Tolmar, Pfizer, Telix, Merck, Serono, Janssen, Bristol‐Myers Squibb, Ipsen, Bayer, Merck Sharpe and Dome, Amgen, and Noxopharm; has received travel and accommodation expenses from Astellas, Merck Serono, Amgen, Novartis, Janssen, Tolmar, and Pfizer; and has received investigator research funding from Astellas, Merck Serono, and AstraZeneca, and institutional research funding from Bristol‐Myers Squibb, AstraZeneca, Aptevo Therapeutics, GlaxoSmithKline, Pfizer, MedImmune, Astellas, Synthorx, Bionomics, Sanofi Aventis, Novartis, Ipsen, Exelixis, Merck Sharpe and Dome, Janssen, Eli Lilly, and Gilead Sciences. Shankar Siva is supported by a Cancer Council Victoria Colebatch Fellowship; has received grants or contracts from Varian, Reflexion, and Bayer Pharmaceuticals; has received honoraria for speakers’ bureaus from AstraZeneca; is a board member for Radiosurgery Society; is a member of the Genitourinary Working Party of the TransTasman Radiation Oncology Group; and is a member of the advanced radiotherapy technology committee for the International Association for the Study of Lung Cancer.

## Provenance

Commissioned; externally peer reviewed.
